# A telehealth approach to central line-associated bloodstream infection prevention activities in nursing homes: the SAFER lines program

**DOI:** 10.1017/ice.2024.203

**Published:** 2025-03

**Authors:** Raveena D. Singh, Bardia Bahadori, Tom Tjoa, Mohamad N. Alsharif, Shereen Nourollahi, Justin Chang, Amarah Mauricio, Jessica Bethlahmy, Syma Rashid, Raheeb Saavedra, Isabel Y. Ashbaugh, Steven Tam, Shruti K. Gohil

**Affiliations:** 1 Division of Infectious Diseases, University of California Irvine, School of Medicine, Irvine, CA, USA; 2 Division of Geriatrics and Gerontology, UC Irvine, School of Medicine, Irvine, CA, USA; 3 Epidemiology & Infection Prevention, UC Irvine Health, Irvine, CA, USA

## Abstract

**Objectives:**

To evaluate the impact of a mobile-app-based central line-associated bloodstream infection (CLABSI) prevention program in nursing home residents with peripherally inserted central catheters (PICCs).

**Design:**

Pre-post prospective cohort study with baseline (September 2015–December 2016), phase-in (January 2017–April 2017), and intervention (May 2017–December 2018). Generalized linear mixed models compared intervention with baseline frequency of localized inflammation/infection, dressing peeling, and infection-related hospitalizations. Cox proportional hazards models compared days-to-removal of lines with localized inflammation/infection.

**Setting:**

Six nursing homes in Orange County, California.

**Patients:**

Adult nursing home residents with PICCs.

**Intervention:**

CLABSI prevention program consisting of an actionable scoring system for identifying insertion site infection/inflammation coupled with a mobile-app enabling photo-assessments and automated physician alerting for remote response.

**Results:**

We completed 8,131 assessments of 817 PICCs in 719 residents (baseline: 4,865 assessments, 422 PICCs, 385 residents; intervention: 4,264 assessments, 395 PICCs, 334 residents). The intervention was associated with 57% lower odds of peeling dressings (OR 0.43, 95% CI 0.28–0.64, *P* < .001), 73% lower local inflammation/infection (OR = 0.27, 95% CI: 0.13–0.56, *P* < .001), and 41% lower risk of infection-related hospitalizations (OR = 0.59, 95% CI: 0.42–0.83, *P* = .002). Physician mobile-app alerting and response enabled 62% lower risk of lines remaining in place after inflammation/infection was identified (HR 0.38, CI: 0.24–0.62, *P* < .001) and 95% faster removal of infected lines from mean (SD) 19 (20) to 1 (2) days.

**Conclusions:**

A mobile-app-based CLABSI prevention program decreased the frequency of inflamed/infected central line insertion sites, improved dressing integrity, increased speed of removal when inflammation/infection were found, and reduced infection-related hospitalization risk.

## Background

Central-line-associated bloodstream infections (CLABSIs) are associated with extensive morbidity, mortality, and substantial excess healthcare costs.^1–3^ National CLABSI prevention efforts have successfully reduced CLABSIs in hospitals.^4,5^ However, the risks of infection from central lines extend beyond hospitalization. Millions of peripherally inserted central catheters (PICCs) are placed each year for care largely managed after hospital discharge.^6–9^ Yet, CLABSI prevention efforts are largely hospital-based and have not yet extended to the 15,000 nursing homes in the United States.^5,9,10^ Strategies to assure high fidelity processes for central line maintenance are poorly defined in this setting, where the volume of patients with complex healthcare needs has outpaced staff training and infection prevention expertise.

CLABSI prevention practices that are relevant to nursing homes are urgently needed. Since most lines are in place before nursing home admission, the mainstay of CLABSI prevention efforts in this setting should focus on the elements of assessment, maintenance, and timely removal. To this end, we evaluated the impact of a CLABSI prevention bundle in nursing homes known as the Standardizing Assessment For Effective Response (SAFER) Lines program consisting of an insertion site score, mobile-app-based photo-assessment and response, and education.

## Methods

We conducted a quasi-experimental cohort study of the SAFER Lines program in adult residents with PICCs at 6 nursing homes in Orange County, California. The study included a baseline observation (September 2015–December 2016), phase-in (January 2017–April 2017), and intervention periods (May 2017–December 2018). Nursing homes were eligible to participate if they admitted adults with PICC lines and facility leadership agreed to implement the SAFER Lines bundle as a Quality Assurance Performance Improvement protocol. This study was approved by the institutional review board of the University of California, Irvine as a minimal risk study; informed consent was waived.

The SAFER Lines program included the use of:

1. **Central Line Insertion Site Assessment (CLISA) score** (Figure 1) which quantifies insertion site inflammation and standardizes erythema by catheter-width (3mm) as a reference. For example, CLISA 1 indicates a radius of erythema <3mm; CLISA 2 3–6 mm erythema; and CLISA 3 >6 mm erythema. Drainage and edema contribute to the score, with presence of pus generating the maximum score of 3. CLISA scores of 2 or 3 indicate localized infection or inflammation. Each score is tied to a recommended response: CLISA 1, warrants watchful waiting; CLISA 2, strong consideration for removal; CLISA 3, urgent removal.
  **Figure 1.**
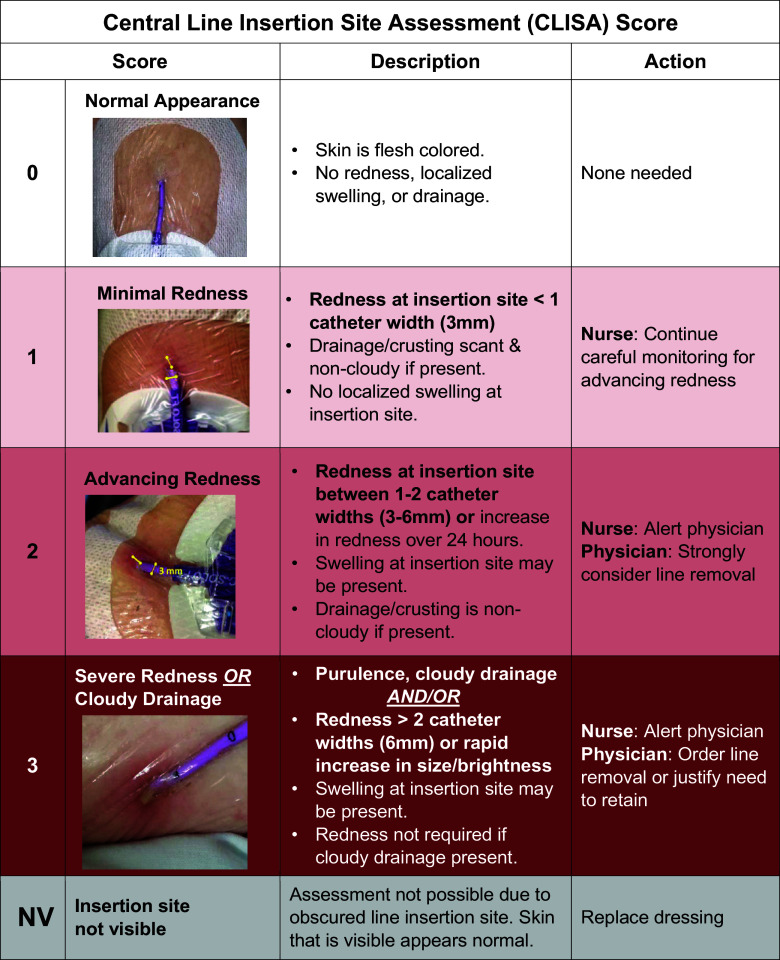
Central line insertion site assessment (CLISA) score provides a framework for assessing and interpreting the presence of localized inflammation or infection at the skin surrounding the insertion site. The width of the catheter size is used to estimate the extent and grade of erythema. Each score is linked with recommended clinician actions, with an expectation to remove central lines with high risk of progression of progression to bloodstream infections (score of 2 or 3).
2. **SAFER Lines mobile-app** that allows daily central line photo-assessments for early detection of high-risk lines and within-app response to prevent progression to CLABSI (Supplemental Figure 1A–B).^11^

### Baseline activities

In the baseline period, research staff conducted daily (Monday–Friday) photo-assessments of PICC line insertion sites and dressings and recorded the presence of localized inflammation or infection using the CLISA scoring system, completing observations without intervention.

### Intervention activities

During phase-in, the SAFER Lines program was implemented as follows:

1. Nurse and physician education on the CLISA score and its expected actions; continued assessment of fever, change in vital signs, and non-visual elements of the skin exam (eg, palpation for tenderness and warmth) was also encouraged.
2. Use of the SAFER Lines mobile-app which enabled (a) daily photo-documentation of insertion sites and CLISA score entry by nurses, (b) automated physicians alerts when high-risk lines were identified (CLISA score 2 or 3, indicating localized inflammation or infection), (c) remote physician examination of current and past insertion site photos, and (d) within-app physician response and ordering for timely response (eg, continue monitoring, remove line, place peripheral IV), Supplemental Figure 1A–B. The app allows nurses to receive physician orders and review whether the provider viewed alerts/messages. If CLISA scores of 2 or 3 were not viewed by physicians within 4 hours, nurses were instructed to send a repeat message through the mobile-app and page/call physicians directly.
3. Dressing maintenance education for nurses (placement, appearance, frequency of changes, and scrub-the-hub practices for line access).

The SAFER Lines mobile-app is a HIPAA-compliant web-based application whereby patient data are encrypted and stored on a centrally secured, firewall-protected webserver at the University of California, Irvine. Nursing homes were given an iPad-Mini for each nursing station; nurses and physicians were trained and individually enrolled with secured username/password logons. Physicians with medical privileges at participating nursing homes downloaded the SAFER Lines mobile-app (MAC and Android OS programs available) onto their own mobile devices.

Research staff visited each nursing home thrice weekly to obtain central line census and record dates of any removed lines. They also remotely monitored nursing photo-assessments and physician responses in real time through a secured webserver where photographs and mobile-app entries were captured and independently assigned CLISA scores; any discrepancies with nursing scores prompted escalation to physician investigators for score verification with nursing feedback/education as needed. Compliance with mobile-app use was monitored and if the number of daily photo-assessments did not match nursing home records for number of lines in place, facility leadership was contacted to encourage completion of missing photo-assessments to assure >95% compliance.

### Data collection

During baseline, research staff collected daily photo-assessments, assigned CLISA scores, and assessed dressing integrity (peeling on one or more sides). Charts were reviewed for documentation of line care or insertion site appearance. Line insertion dates were recorded as documented in physician, nursing, or procedure notes; if unavailable, the first documented date of line presence from chest radiograph was used and if that was unavailable, nursing home transfer date from hospital was used. Line removal dates/times were recorded per chart documentation and confirmed verbally with nurses or by visual confirmation of line presence on the last date of assessment by research staff who were on site daily on weekdays.

During intervention, nurses completed daily photo-assessments using the SAFER Lines mobile-app as described above, entering CLISA scores, dressing integrity, resident admission date, line insertion, and removal dates/times; dates were confirmed by research staff through chart review and verbal verification with nursing staff as needed. Nursing and physicians’ mobile-app entries were captured electronically.

Finally, we assessed infection-related hospitalizations and bacteremia among nursing home residents during the baseline and intervention periods by linking nursing home data from the Centers for Medicaid & Medicare Services’ Minimum Data Set with state hospitalization data.^12,13^ Infections were defined using hospital International Statistical Classification of Diseases and Related Health Problems, 10th Revision (ICD-10) codes as a primary or secondary diagnoses with a present-on-admission indicator.^14^

### Outcomes

Outcomes were (1) CLISA 2, CLISA 3, and the composite of CLISA 2 or 3 as indicators of localized inflammation or infection, (2) days-to-line removal from first CLISA 2 or 3 (composite) and the subsets of CLISA 2 and CLISA 3, (3) presence of any peeling on dressings, and (4) discharge from the nursing home to a hospital due to an infection.

### Statistical analysis

Residents were described by age, sex, and history of prior PICC (as available using line insertion and removal dates within the study cohort). PICC line characteristics and dressing integrity were evaluated per line and across assessments. Data from phase-in were not included in any analyses. Lines were described by frequency, dwell time (summed days from insertion to removal), maximum CLISA scores recorded during a line’s duration (each line was assigned into a mutually exclusive category), and days-to-line removal (summed days from first maximum CLISA score to date of line removal). Lines with insertion sites that could not be visualized in any assessment were not included in outcome analysis (eg, covered by gauze or antibacterial-disc). Lines without removal dates were not included in dwell time or days-to-line removal calculations. We additionally assessed the proportion of lines with dressings with any peeling. Chi-square tests compared baseline and intervention proportions of lines with abnormal CLISA scores, peeling dressings, hospitalization, and bacteremia events. T-tests compared mean dwell time and days-to-line removal after CLISA score of 2 or 3.

Generalized linear mixed-effects (GLIMMIX) logistic regression models clustering by resident and facility (random intercepts) evaluated the effect of the SAFER Lines program on the following outcomes: (1) CLISA 2, CLISA 3, and composite CLISA 2 or 3 in separate models with independent variables of study period, age (years), sex, line dwell time (days), dressing peeling on one or more sides, and history of previous PICC line; (2) proportion of lines with peeling dressings adjusting for age, sex, line dwell time, and history of previous line; and (3) proportion of residents hospitalized due to an infection adjusting for age, gender, line dwell time, dressing peeling, and history of previous line. Kaplan–Meier curves were generated for the duration that lines were retained with an abnormal CLISA score; Cox proportional hazards models (adjusted for age, gender, and history of prior line) were used to evaluate the effect of the intervention on these outcomes while accounting for clustering (variance correction) at the patient level. Additionally, we performed a sensitivity analysis of all outcomes after removing the facility that did not participate in the intervention period. All analyses were completed using SAS version 9.4 (Cary, NC).

## Results

Six nursing homes participated. Due to changes in administrative leadership, one facility discontinued participation during the phase-in period and another had a 10-month delay in intervention implementation (completing 9 of 19 months). Table 1 summarizes nursing home and resident characteristics. There were 4,865 assessments of 422 PICCs in 385 nursing home residents during baseline and 4,264 assessments of 395 PICCs in 334 residents during intervention. Of these, 69.0% (291/422) and 82.0% (324/395) of PICCs had visible insertion sites (not covered by gauze or antibacterial-disc) during baseline and intervention, respectively.

**Table 1.** Characteristics of participating nursing homes and residents with peripherally inserted central catheters

	Baseline N (%) 15 months	Intervention N (%) 19 months	*P*-value
Nursing Home Characteristics, median (range)			
Facilities (N)	6	5	
Licensed beds	99 (99–174)	99 (97–169)	
Annual admissions	664 (277–964)	640 (325–805)	
Daily census	94 (88–147)	88 (77–140)	
Length of stay, days	206 (183–221)	209 (199–216)	
Resident Characteristics^a^			
Residents with central lines (N)	385	334	
Age in years (mean, SD)	67 (15)	67 (16)	.648
Male sex	184 (48.2)	138 (48.8)	.879
Prior central line	31 (8.1)	46 (13.8)	.013
Total number of PICC lines	422	395	
Assessments completed	4,865	4,264	.921
Dwell time in days (mean, SD)	32 (36.8)	23 (25.0)	<.001

a Nursing home residents with peripherally inserted central catheter (PICC) line in place.

### Baseline observations

At baseline, 40.9% (119/291) of lines had dressings with peeling on one or more sides, 29.9% (87/291) had at least one assessment with a CLISA 2 or 3 and once identified, mean days to removal was 20 (SD = 19) days, Table 2. Supplemental Figures 2–3 illustrate examples of dressing maintenance and insertion site findings we encountered. In 8 separate patients, research staff were ethically compelled to break research protocol of non-intervention and escalate their concern to medical directors when residents had severely symptomatic insertion sites (pain at the insertion site, frank purulence or abscess) unrecognized by clinical teams.

**Table 2.** Dressing integrity, presence of insertion site inflammation or infection, and days-to-line removal during baseline and intervention periods

	Baseline N (%) 15 months	Intervention N (%) 19 months	*P*-Value^a^
PICC Lines with Visible Insertion Sites^**b**^	291 (69.0)	324 (82.0)	<.001
PICC Line Dwell Time (mean, SD)	37.1 (36.8)	26.2 (25.8)	<.001
Dressings Peeling on ≥1 Side^**c**^	119 (40.9)	66 (20.4)	<.001
Maximum CLISA Score^**c**,d^			
CLISA 0	107 (36.8)	88 (27.2)	<.001
CLISA 1	97 (33.3)	197 (60.8)	
CLISA 2	54 (18.6)	30 (9.3)	
CLISA 3	33 (11.3)	9 (2.8)	
CLISA 0 or 1 (composite)	204 (70.1)	285 (88.0)	<.001
CLISA 2 or 3 (composite)	87 (29.9)	39 (12.0)	<.001
Days-to-Line Removal^e^			
From first CLISA 2 (mean, SD)	20 (18)	13 (22)	.1
From first CLISA 3 (mean, SD)	19 (20)	1 (2)	<.001
From first CLISA 2 or 3 (mean, SD)	20 (19)	10 (19)	.01
All Visible Assessments^f^	2,243	3,266	
CLISA 0	1,248 (55.6)	1,875 (57.4)	<.001
CLISA 1	609 (27.2)	1,317 (40.3)	
CLISA 2	303 (13.5)	60 (1.8)	
CLISA 3	83 (3.7)	14 (0.4)	

a *P*-value calculated using χ2 for dressing peeling and CLISA scores; *t*-test used for dwell time and days to removal.

b Denominator for percentage is total number of lines: n = 422 during baseline and n = 395 during intervention.

c Denominator for percentages is number of PICC lines with visible insertion sites.

d Mutually exclusive categories with each line assigned according to maximum CLISA (central line insertion site assessment) score through line duration.

e Calculated using date of first maximum CLISA score to date of line removal; for lines without a known removal date, the last day of line assessment was used.

f Denominator for percentages presented for each subcategory below.

### Changes in central line maintenance practices observed after implementing the SAFER lines program

Though the main target of the SAFER Lines program was to monitor insertion sites for CLISA 2 or 3 and remove lines in a timely manner, we observed improvements in all maintenance activities. Comparing baseline to intervention, there was a 50% decrease in lines with peeling dressings, from 40.9% to 20.4%;in adjusted analyses, the SAFER Lines program was associated with a 57% lower odds of having a dressing with peeling (OR 0.43, 95% CI 0.28–0.64, *P* < .001), Table 3. Results remained significant on sensitivity analysis (OR = 0.47, 95% CI: 0.31–0.74, *P* < .001).

**Table 3.** Multivariable model: impact of the SAFER lines CLABSI prevention bundle on proportion of lines with localized inflammation or infection^a,b^

Variables	Local Inflammation or Infection at Insertion Site					
	CLISA Score 2 or 3		CLISA Score 2		CLISA Score 3	
	Odds Ratio (CI)	*P*-Value	Odds Ratio (CI)	*P*-Value	Odds Ratio (CI)	*P*-Value
SAFER Lines Intervention^**c**^	0.27 (0.13–0.56)	<.001	0.35 (0.16–0.75)	.008	0.27 (0.12–0.64)	.003
Age	0.99 (0.98–1.01)	.43	0.99 (0.97–1.01)	.47	1.00 (0.98–1.03)	.98
Male sex	1.78 (1.03–3.09)	.04	1.55 (0.82–2.95)	.18	1.79 (0.88–3.66)	.11
History of prior central line	1.70 (0.83–3.45)	.14	2.06 (0.88–4.81)	.09	0.96 (0.38–2.42)	.94
Dressing peeling on ≥1 side	2.15 (1.17–3.94)	.01	1.44 (0.73–2.84)	.28	3.05 (1.43–6.51)	.004
PICC line dwell time (days)	1.01 (0.99–1.01)	.16	1.00 (0.99–1.02)	.44	1.01 (1.00–1.02)	.23

a Results are based on generalized linear mixed effects logistic regression models done at the PICC line level, accounting for clustering (random intercepts) within nursing homes and patients.

b SAFER, Standardizing Assessments For Effective Response; CLABSI, central line associated bloodstream infection.

c Results were unchanged after sensitivity analysis removing the facility that did not complete the intervention period: CLISA 2 or 3 – OR 0.26, 95% CI 0.12–0.56, *P* < .001; CLISA 2 – OR 0.35, 95% CI 0.16–0.77, *P* = .009; CLISA 3 – OR 0.26, 95% CI 0.11–0.61, *P* = .002).

During baseline, there was heavy use of gauze and other materials covering the insertion site and the proportion of assessments with dressings where insertion sites were not visible decreased from 2,313 to 998 during the baseline and intervention periods, respectively. Daily nursing documentation of dressing and insertion site appearance improved from <1% during baseline to >98% in the last month of intervention.

### Changes in CLISA score observed after implementing the SAFER lines program

Among lines with at least one assessment of a visible insertion site, the proportion with localized infection or inflammation (CLISA 2 or 3) decreased from 29.9% (87/291) during baseline to 12.0% (39/324) during the intervention (59.9% reduction, *P* < .001), Table 2. On adjusted analyses (Table 3), the intervention was associated with 73% lower odds of developing CLISA 2 or 3, OR = 0.27 (95% CI 0.13–0.56), *P* < .001. Results remained significant on sensitivity analyses (Table 3 footnotes). Similar results were found for the separate subsets of CLISA 2 and 3. Notably, peeling dressings were significantly associated with development of CLISA 3, adjusted OR = 3.05 (95% CI: 1.43–6.51), *P* = .004 (Table 3).

### Changes in days-to-line removal after CLISA 2 or 3 upon implementing the SAFER lines program

Line removal after CLISA 2 or 3 occurred 50% faster, from a mean (SD) of 20 (19) days during baseline to 10 (19) days after intervention (*P* = .01), Table 2. In the subset of lines with CLISA 3, removal was 95% faster, from 19 (20) days to 1 (2) day (*P* < .001) during the baseline versus intervention periods, respectively. Mean PICC dwell time also decreased 38% from 37.1 (36.8) days to 26.3 (25.8) days.

**Figure 2.**
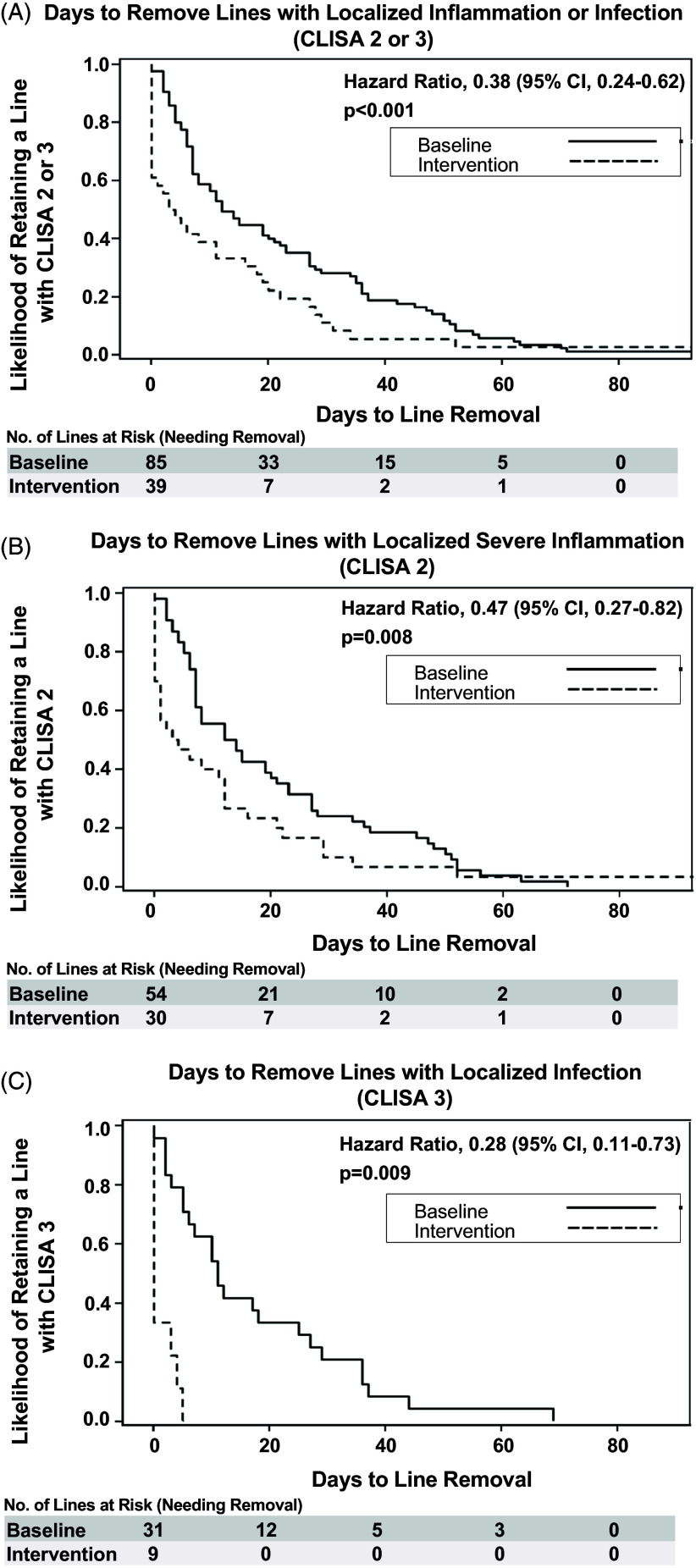
Probability of removal of lines identified with inflammation or infection during the baseline versus intervention periods. **(A–C):** Kaplan–Meier curves for estimated probability of line removal when localized inflammation or infection are identified according to **(1A)** CLISA (central line insertion site assessment) scores of 2 or 3, composite of localized inflammation or infection; **(1B)** CLISA score 2 indicating progressive localized inflammation **(1C)** CLISA score 3 indicating severe inflammation or infection (severe erythema or purulence). Cox proportional hazards models to evaluate days-to-removal for baseline and intervention periods adjusted for adjusted for age, gender, and history of prior line. Results remained unchanged on sensitivity analyses after removing the facility that did not complete the intervention period as follows: CLISA 2 or 3—hazard ratio (HR) 0.40, 95% CI 0.25–0.65, *P* < .001; CLISA 2—HR 0.52, 95% CI 0.29–0.93, *P* = .026; CLISA 3—HR 0.26, 95% CI 0.10–0.68, *P* = .006).

The SAFER Lines program was associated with 62% lower risk of remaining in place once CLISA 2 or 3 was identified [HR = 0.38 (95% CI: 0.24–0.62), *P* < .001] and a 72% lower risk of remaining in place once a CLISA 3 was identified [HR = 0.28 (95% CI: 0.11–0.73), *P* = .009]. Results remained significant on sensitivity analyses (Figure 2, caption). Kaplan–Meier curves for days-to-line removal upon first identification of CLISA 2 or 3 are shown for baseline and intervention periods in Figures 2A–C.

### Infection-related hospitalization and bacteremia

The proportion of nursing home residents who were discharged to a hospital due to an infection decreased by 27.3% from 42.9% (165/385) to 31.1% (104/334) during the intervention and baseline periods, respectively. On adjusted analyses, the SAFER Lines program was associated with a 41% lower odds of an infection-related hospitalization, OR = 0.59 (95% CI, 0.42–0.83), *P* = .002. Results remained significant on sensitivity analyses (OR = 0.58, 95% CI: 0.41–0.82, *P* = .002). The proportion of residents hospitalized with bacteremia decreased by 48.8% from 2.3% (9/385) to 1.2% (4/334) during the baseline and intervention periods, respectively, (*P* = .27).

## Discussion

Effective CLABSI prevention strategies are urgently needed to address the increasing numbers of patients who require intravenous therapy outside of hospitals. Obstacles to effective CLABSI prevention in the nursing home include high staff turnover, insufficient nurse-to-patient ratios, lack of infection prevention training or expertise, and lack of onsite physicians for proactive responses.^15–20^ These systemic issues predispose staff to breakdowns in basic practices including dressing maintenance, monitoring, and recognition of skin changes. Before our intervention, we found that failures in appropriate line care were exceedingly common: almost no lines had documentation of visual findings, 40% had peeling dressings, nearly 20% had serious local inflammation or infection, and 30% of lines experienced serious local inflammation or infection before discontinuation.

Importantly, we found that peeling dressings were associated with a 2 to 3-fold higher risk of insertion site inflammation or infection (Table 3), providing new evidence for the importance of maintaining dressing integrity in preventing localized infection. Even when CLISA 2 or 3 lines were identified, mean time to removal was 20 days, leaving lines high risk for CLABSI in place for weeks.

The SAFER Lines CLABSI prevention bundle was associated with multiple improvements in line care. First, dressing maintenance improved, including a 50% increase in intact dressings and near perfect adherence with documenting visual assessment. Second, insertion site inflammation or infection was reduced by three-quarters, and removal of CLISA 2 or 3 lines occurred twice as quickly, with the subset of CLISA 3 lines being removed in less than 2 days. Finally, we found a 27% lower rate of hospitalization due to infection.

The success of the SAFER Lines program was likely due to its ability overcome several operational barriers. By providing a common language and standardized metrics to identify localized infection with associated expectations for action, the CLISA score facilitated early identification of high-risk lines and timely response.^11^ Moreover, physician auto-alerting for CLISA 2 or 3 allowed remote examinations of serial photos over time, which primed physicians with all the necessary information to place orders within the app, which facilitated timely action. This approach dramatically reduced the response time to high-risk local symptoms from nearly 2 weeks to less than 1 day.

Notably, enforcement of daily documentation may also have prompted evaluation of line necessity. Some nurses reported that they had started to prompt physicians for removal of PICC lines that were no longer being used. This was supported by a reduction in overall line dwell time by nearly 40%.

Our intervention is an example of a successful telemedicine approach to facilitate infection prevention. The mobile-app allowed meaningful communication between doctors and nurses about non-emergent findings and encouraged proactive measures for prevention that might otherwise go undiscussed. Nurses in nursing homes usually reserve phone calls or pages to physicians for events that require immediate diagnostics or treatments and otherwise await regularly planned physician visits for additional orders. For central line-associated events, the usual trigger to contact a treating physician would be fever or acute pain at the line. By providing a way to alert physicians with high-yield information about a high-risk line through a mobile-app, nurses could garner physician attention before severe symptoms arose.

Our study has several important limitations. First, while we were able to demonstrate statistically significant reductions in infection-related hospitalizations using coded diagnosis data and also observed a nonsignificant decrease in bacteremia by nearly 50%, our sample size was insufficient to evaluate bacteremia or CLABSI events. Second, CLISA evaluation was limited to only insertion sites that were visible, which impacted our sample size. Nevertheless, training increased the proportion of visible insertion sites as the intervention taught nurses that localized inflammation or infection provides a useful clinical end point since extraluminal introduction of pathogens is a known pathway for the development of CLABSI.^21,22^ Despite sample size limitations, we saw dramatic, statistically significant improvements in CLISA scores. Third, we are unable to differentiate the relative impact of the mobile-app versus education in our bundled intervention. Fourth, our quasi-experimental design precludes determination of causality. Finally, erythema can be difficult to visualize in patients with darker skin tones on both clinician and photo-assessments.^23–25^ Though understudied, there is growing recognition that patients with darker skin tones are at higher risk for unrecognized skin/soft tissue infections and late presentation with sepsis, higher morbidity, and mortality.^26–28^ Solutions to detect infection earlier in darker pigmented individuals are urgently needed. In our cohort, photo-assessments could capture purulence (CLISA 3) and dressing maintenance targets (peeling and soiling) in residents with darker skin tones.

The SAFER Lines program successfully improved dressing quality, reduced localized inflammation and infection at PICC insertion sites, facilitated rapid removal of lines at high risk for progression to CLABSI, and reduced infection-related hospitalizations in nursing home residents. This work provides evidence supporting a new strategy for CLABSI prevention that is tailored to the needs of the nursing home setting and highlights the gains that can be achieved through photo-assessments and mobile-app-based approaches.

## Supporting information

Singh et al. supplementary material Singh et al. supplementary material

## Supplementary material

The supplementary material for this article can be found at https://doi.org/10.1017/ice.2024.203

## References

[1] Khong CJ , Baggs J , Kleinbaum D , Cochran R , Jernigan JA. The likelihood of hospital readmission among patients with hospital-onset central line-associated bloodstream infections. Infect Control Hosp Epidemiol 2015;36:886–892. 25990620 10.1017/ice.2015.115 PMC4702248

[2] Stevens V , Geiger K , Concannon C , Nelson RE , Brown J , Dumyati G. Inpatient costs, mortality and 30-day re-admission in patients with central-line-associated bloodstream infections. Clin Microbiol Infect 2014;20:24–318. 10.1111/1469-0691.12407 24112305

[3] Centers for Disease C, Prevention. Vital signs: central line-associated blood stream infections--United States, 2001, 2008, and 2009. MMWR Morb Mortal Wkly Rep 2011;60:243–248. 21368740

[4] Marschall J , Mermel LA , Fakih M , et al. Strategies to prevent central line-associated bloodstream infections in acute care hospitals: 2014 update. Infect Control Hosp Epidemiol 2014;35:753–771. 24915204 10.1086/676533

[5] Buetti N , Marschall J , Drees M , et al. Strategies to prevent central line-associated bloodstream infections in acute-care hospitals: 2022 update. Infect Control Hosp Epidemiol 2022;43:553–569. 35437133 10.1017/ice.2022.87 PMC9096710

[6] Chopra V , Montoya A , Joshi D , et al. Peripherally inserted central catheter use in skilled nursing facilities: a pilot study. J Am Geriatr Soc 2015;63:1894–1899. 26312402 10.1111/jgs.13600 PMC4626207

[7] Harrison JM , Dick AW , Stone PW , et al. Infection trends in home health care, 2013–2018. Infect Control Hosp Epidemiol 2021;42:1388–1390. 34766902 10.1017/ice.2021.248 PMC8931666

[8] Shang J , Wang J , Adams V , Ma C. Risk factors for infection in home health care: analysis of national outcome and assessment information set data.Res Nurs Health 2020;43:373–386. 32652615 10.1002/nur.22053 PMC7418221

[9] Keller SC , Williams D , Rock C , Deol S , Trexler P , Cosgrove SE. A new frontier: central line-associated bloodstream infection surveillance in home infusion therapy. Am J Infect Control 2018;46:1419–1421. 29908838 10.1016/j.ajic.2018.05.016 PMC6329378

[10] Spires SS , Rebeiro PF , Miller M , Koss K , Wright PW , Talbot TR. Medically attended catheter complications are common in patients with outpatient central venous catheters. Infect Control Hosp Epidemiol 2018;39:439–444. 29444733 10.1017/ice.2018.8 PMC5972823

[11] Gohil SK , Yim J , Quan K , et al. Impact of a central-line insertion site assessment (CLISA) score on localized insertion site infection to prevent central-line-associated bloodstream infection (CLABSI). Infect Control Hosp Epidemiol 2020;41:59–66. 31699181 10.1017/ice.2019.291

[12] Centers for Medicare & Medicaid Services. Minimum Data Set (MDS) 3.0 for Nursing Homes and Swing Bed Providers. https://www.cms.gov/Medicare/Quality-Initiatives-Patient-Assessment-Instruments/NursingHomeQualityInits/NHQIMDS30. Accessed March 1, 2023.

[13] California Office of Statewide Health Planning and Development. California Department of Health Care Access and Information. https://hcai.ca.gov/. Accessed August 1, 2023.

[14] Agency for Healthcare Research Quality. Patient Safety Index ICD-10, 2022. Healthcare Cost and Utilization Project (HCUP). https://qualityindicators.ahrq.gov/measures/psi_resources. Published 2022. Accessed January 20, 2024.

[15] Harrington C , Kovner C , Mezey M , et al. Experts recommend minimum nurse staffing standards for nursing facilities in the United States. Gerontologist 2000;40:5–16. 10750309 10.1093/geront/40.1.5

[16] Kovner CT , Mezey M , Harrington C. Who cares for older adults? Workforce implications of an aging society. Health Aff (Millwood) 2002;21:78–89. 10.1377/hlthaff.21.5.78 12224911

[17] Kim H , Kovner C , Harrington C , Greene W , Mezey M. A panel data analysis of the relationships of nursing home staffing levels and standards to regulatory deficiencies. J Gerontol B Psychol Sci Soc Sci 2009;64:269–278. 19181692 10.1093/geronb/gbn019 PMC2655170

[18] Zimmerman S , Gruber-Baldini AL , Hebel JR , Sloane PD , Magaziner J. Nursing home facility risk factors for infection and hospitalization: importance of registered nurse turnover, administration, and social factors. J Am Geriatr Soc 2002;50:1987–1995. 12473010 10.1046/j.1532-5415.2002.50610.x

[19] Smith PW , Bennett G , Bradley S , et al. SHEA/APIC guideline: infection prevention and control in the long-term care facility. Am J Infect Control 2008;36:504–535. 18786461 10.1016/j.ajic.2008.06.001 PMC3375028

[20] Cadogan MP , Franzi C , Osterweil D , Hill T. Barriers to effective communication in skilled nursing facilities: differences in perception between nurses and physicians. J Am Geriatr Soc 1999;47:71–75. 9920232 10.1111/j.1532-5415.1999.tb01903.x

[21] Mermel LA. What is the predominant source of intravascular catheter infections? Clin Infect Dis 2011;52:211–212. 21288845 10.1093/cid/ciq108

[22] Edgeworth J. Intravascular catheter infections. J Hosp Infect 2009;73:323–330. 19699555 10.1016/j.jhin.2009.05.008

[23] Johnson J , Johnson AR Jr , Andersen CA , Kelso MR , Oropallo AR , Serena TE. Skin pigmentation impacts the clinical diagnosis of wound infection: imaging of bacterial burden to overcome diagnostic limitations. J Racial Ethn Health Disparities 2024;11:1045–1055. 37039975 10.1007/s40615-023-01584-8 PMC10933203

[24] Oozageer Gunowa N. Skin tone bias and wound care: highlighting the current evidence and addressing the gaps in knowledge of dark skin tones. Wounds UK 2022;18:22–27.

[25] Frew J , Penzi L , Suarez-Farinas M , et al. The erythema Q-score, an imaging biomarker for redness in skin inflammation. Exp Dermatol 2021;30:377–383. 33113259 10.1111/exd.14224 PMC8049083

[26] Pusey-Reid E , Quinn L , Samost ME , Reidy PA. Skin assessment in patients with dark skin tone. Am J Nurs 2023;123:36–43. 10.1097/01.NAJ.0000921800.61980.7e 36815818

[27] Ray GT , Suaya JA , Baxter R. Incidence , microbiology, and patient characteristics of skin and soft-tissue infections in a U.S. population: a retrospective population-based study. BMC Infect Dis 2013;13:252. 23721377 10.1186/1471-2334-13-252 PMC3679727

[28] Arif N , Yousfi S , Vinnard C. Deaths from necrotizing fasciitis in the United States, 2003–2013. Epidemiol Infect 2016;144:1338–1344. 26548496 10.1017/S0950268815002745 PMC5725950

