# Supplementary material for: A telehealth approach to central line-associated bloodstream infection prevention activities in nursing homes: the SAFER lines program
Source: Infect Control Hosp Epidemiol. 2025 Feb 3;46(3):236–42. doi: 10.1017/ice.2024.203 (PMC11883653; doi:10.1017/ice.2024.203)
Supplement: Singh et al. supplementary material [file S0899823X24002034sup001.docx]

**Supplemental Tables and Figures**

**Supplemental Figure 1**

**
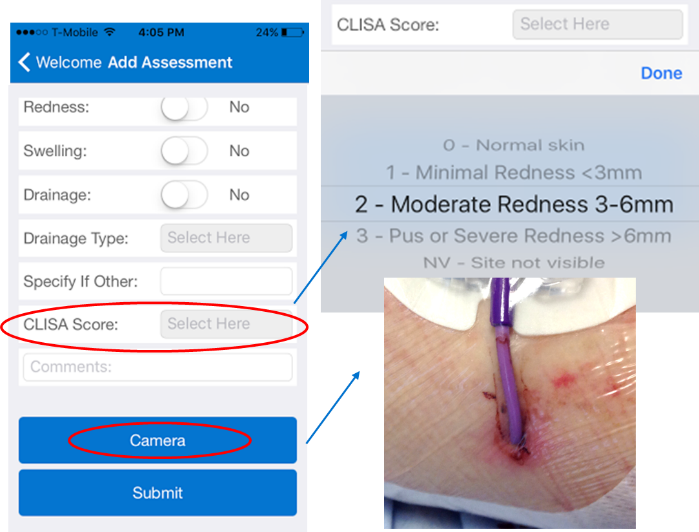

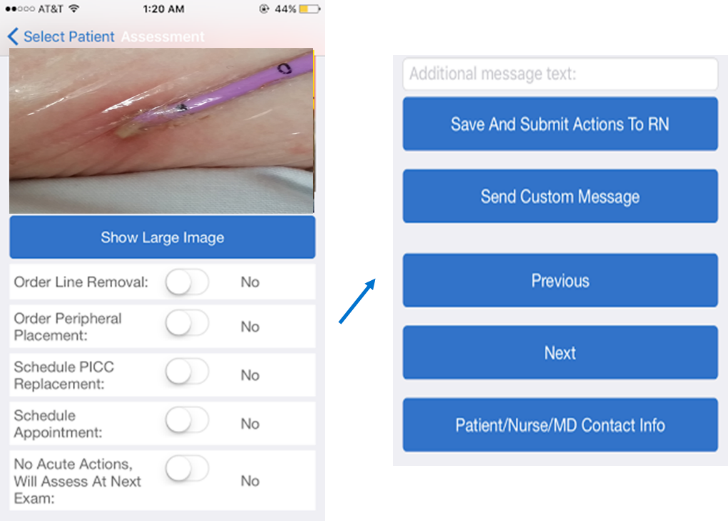
**

**B. Physician Response Within Mobile-App**

**A. Mobile-App Nursing Photo-Assessments**

**Supplemental Figure 1A-B: The Standardized Assessments For Effective Response (SAFER) Lines Mobile App**: The SAFER Lines mobile app was designed to allow remote clinician monitoring of central line insertion sites and response when high risk central lines are found. **(A)** Nurses use the mobile-app to take photo-assessments and record the central line insertion site assessment (CLISA) score. (B) Physicians receive alerts when a high risk CLISA score of 2 or 3 are identified, enabling remote examination of the insertion and within-app ordering of appropriate actions. All data are stored on a secured web-based platform (not on the mobile device).

**Supplemental Figure 2**


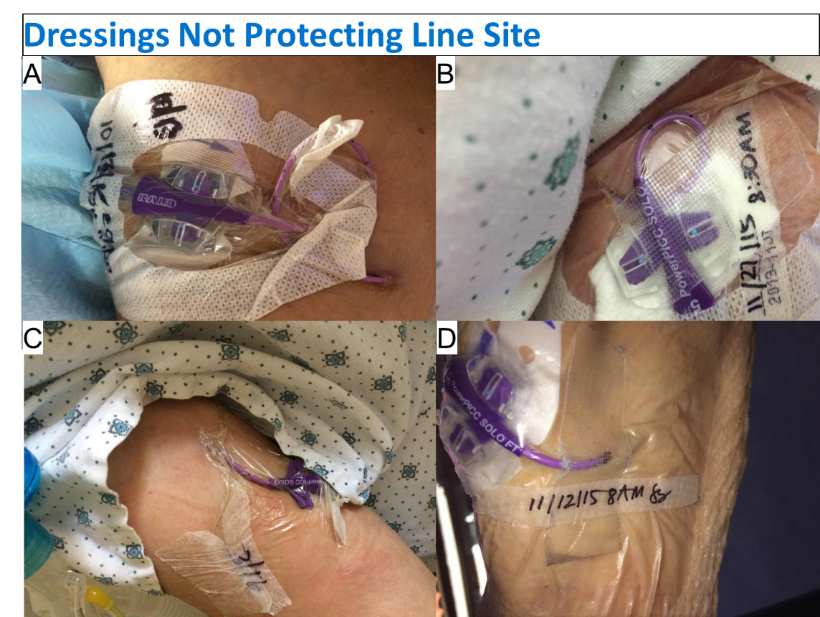


**Supplemental Figure 2 –** Dressings not protecting the insertion site: (A) Dressing peeling with full exposure of insertion site. (B) PICC hub placed on top of gauze and tape used on top of the dressing, disrupting dressing permeability, (C) Dressing peeling and use of a non-bordered dressing, no securement device. (D) Tape place on top of dressing, line angled to the side of arm (not in anatomic line with brachial vein) causing tension of skin at insertion site, use of non-bordered dressing.

**Supplemental Figure 3**


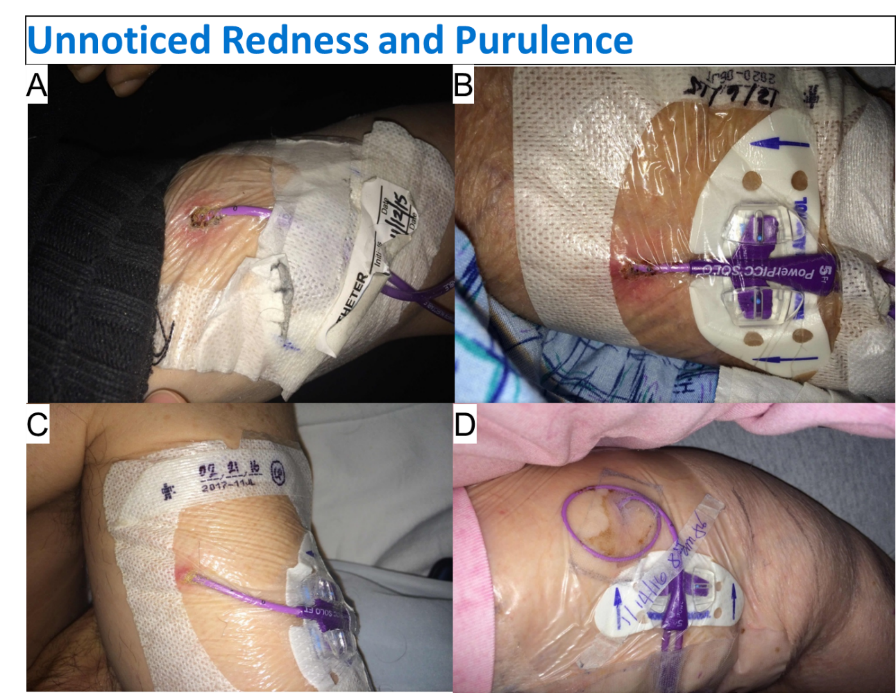


**Supplemental Figure 3 –** Localized redness and purulence unrecognized and without clinician response during baseline. (A) Redness, ulceration, drainage at insertion site. (B) Redness, edema, possible localized abscess at insertion site, (C) Purulent drainage at insertion site. (D) Cloudy serosanguinous drainage ongoing for days before dressing was changed.
